# Virtual Reality for Decreasing Procedural Pain during Botulinum Toxin Injection Related to Spasticity Treatment in Adults: A Pilot Study

**DOI:** 10.3390/medicina60010023

**Published:** 2023-12-22

**Authors:** Romain David, Alexis Dumas, Etienne Ojardias, Solène Duval, Amine Ounajim, Anaïck Perrochon, Carlos Luque-Moreno, Maarten Moens, Lisa Goudman, Philippe Rigoard, Maxime Billot

**Affiliations:** 1 PRISMATICS Lab (Predictive Research in Spine/Neuromodulation Management and Thoracic Innovation/Cardiac Surgery), Poitiers University Hospital, 86000 Poitiers, France; amine.ounajim@chu-poitiers.fr (A.O.);; 2 Physical and Rehabilitation Medicine Unit, Poitiers University Hospital, University of Poitiers, 86000 Poitiers, France; 3 Physical Medicine and Rehabilitation Department, University Hospital of Saint-Etienne, 42270 Saint-Etienne, France; 4 Lyon Neuroscience Research Center, Trajectoires Team, Inserm UMR-S 1028, CNRS UMR 5292, Lyon1 and Saint-Etienne Universities, 42270 Saint-Etienne, France; 5 HAVAE, UR20217, University of Limoges, F-87000 Limoges, France; anaick.perrochon@unilim.fr; 6 Instituto de Biomedicina de Sevilla, IBiS, Departamento de Fisioterapia, Universidad de Sevilla, 41009 Seville, Spain; carloslm@us.es; 7 Department of Neurosurgery, Universitair Ziekenhuis Brussel, Laarbeeklaan 101, 1090 Brussels, Belgium; 8 STIMULUS Consortium (Research and Teaching Neuromodulation uz Brussel), Vrije Universiteit Brussel, Laarbeeklaan 103, 1090 Jette, Belgium; 9 Department of Radiology, Universitair Ziekenhuis Brussel, Laarbeeklaan 101, 1090 Brussels, Belgium; 10 Research Foundation—Flanders (FWO), 1090 Brussels, Belgium; 11 Department of Neuro-Spine Surgery & Neuromodulation, Poitiers University Hospital, 86000 Poitiers, France; 12 Prime Institute UPR 3346, CNRS, ISAE-ENSMA, University of Poitiers, 86000 Poitiers, France

**Keywords:** central nervous system disease, complementary therapies, virtual reality, pain, anxiety

## Abstract

Background and Objectives: Botulinum toxin injections are commonly used for the treatment of spasticity. However, injection procedures are associated with pain and procedural anxiety. While pharmacological approaches are commonly used to reduce these, innovative technology might be considered as a potential non-pharmacological alternative. Given this context, immersive virtual reality (VR) has shown effectiveness in the management of procedural pain. Our retrospective pilot study aimed to assess the potential added value of virtual reality in the management of pain and anxiety during intramuscular injections of botulinum toxin. Materials and Methods: Seventeen adult patients receiving botulinum toxin injections were included. A numerical rating scale was used to assess pain and anxiety during the injection procedure. The patients reported the pain experienced during previous injections without VR before injection and the pain experienced in the current procedure with VR after the end of the procedure. The level of satisfaction of VR experience, whether or not they agreed to reuse VR for the subsequent toxin botulinum injection, and whether or not they would recommend VR to other patients were assessed. Results: The use of virtual reality led to a decrease of 1.8 pain-related points compared to the procedure without technology. No significant improvement in the level of anxiety was reported. Patients were very satisfied with their VR experiences (7.9 out of 10), and many would agree to reuse VR in their next injection procedure (88%) and to recommend the use of VR in other patients (100%). Conclusion: VR was useful for managing procedural pain related to botulinum toxin injection in adults, with a high level of satisfaction reported by the patients. VR should be considered as a valuable alternative to pharmacological approaches to manage procedural pain during botulinum toxin injection in adults.

## 1. Introduction

Neurological impairments such as stroke, multiple sclerosis, and cerebral palsy represent a major public health issue worldwide [1]. Motor impairments associated with these pathologies, and more specifically, spasticity, are associated with loss of mobility and social participation [2]. Spasticity is defined as a motor disorder characterized by a velocity-dependent increase in the tonic stretch reflexes (muscle tone) with exaggerated tendon jerks, resulting from hyperexcitability of the stretch reflexes as one component of upper motor neuron (UMN) syndrome [3]. Intramuscular botulinum toxin injection is a first-line treatment for the management of spasticity in adult patients [4]. Toxin injections help to reduce disturbing muscle hypertonia and consequently improve functional capacities, relieve pain related to spasticity, enhance hygiene gesture capabilities, and improve quality of life [4,5]. While the literature provides strong evidence of the clinical benefits of botulinum toxin, the injection procedure is associated with procedural pain and anxiety [6,7,8].

Several factors influence procedural pain, such as the use of an anesthetic at the injection site [9] or the method of injection site location (ultrasound or electrostimulation) [7,10]. Regardless of the type of procedure, pain is still reported by patients [10], and repetitions of injection exacerbate pain symptoms [11]. Ultimately, pain could lead to discontinuation of the injection process, representing a loss of chance for the patient [12]. In this context, pain should be managed with the view of improving treatment adherence and optimizing therapy. 

When procedural pain is reported by the patient as problematic, pharmacological approaches, such as local anesthetic cream of lidocaine/prilocaine (EMLA^®^) or systemic therapeutics like MEOPA and midazolam, have shown clinical efficacy in relieving pain and discomfort during botulinum toxin injection [4,12,13,14,15,16,17]. However, these drugs have been associated with adverse effects, including sleepiness, nausea, and dizziness [15,18]. 

As a non-pharmacological complementary, innovative technology such as virtual reality (VR) has recently been introduced in different departments to manage pain (in acute, chronic and experimental settings) [19,20,21,22,23]. VR is defined by a computerized system that creates a virtual environment where a person undergoes an immersive sensory experience with an enriched environment involving multiple augmented sensory feedbacks (auditory, visual, and tactile enriched VR environment) [24]. VR has the advantage of being easy to use, quick to set up, being accessible with very little training, not requiring any supplementary staff and having few non-serious undesirable effects (0 to 8% nausea and dizziness) [25]. The immersive environment is reinforced by combining audio guidance with display of an appeasing visual scene. VR has proven its effectiveness in the management of procedural pain [26], particularly pain associated with venipuncture [25,27]. In a retrospective chart review, Chau et al. [28] showed the feasibility of using VR during botulinum toxin injections in 14 pediatric patients and reported benefits in the management of procedural pain [28]. In adults, VR also seems to offer advantages in some hospital settings, even in other types of injections, regarding pain, anxiety, and anger management due to the distraction provided by this technology [29,30,31]. However, the effects and feasibility of VR during the intramuscular injection of botulinum toxin in adults presenting with spasticity have yet to be determined. 

The main objective of our study was to assess VR’s efficacy in the management of procedural pain during intramuscular injections of botulinum toxin in adult patients presenting with spasticity. Based on the literature, we hypothesized that, in comparison with non-VR, VR would induce a decrease in procedural pain. The secondary objectives were to determine the potential efficacy of VR in alleviating anxiety and assessing the level of patient satisfaction. 

## 2. Materials and Methods

### 2.1. Study Design

This was a retrospective study conducted at the Physical Medicine and Rehabilitation Department of the University Hospital of Poitiers between February and August 2022. Data collection was conducted according to the guidelines of the Declaration of Helsinki and the French Data Protection Authority (CNIL, MR-004). All data collection was declared to Health Data Hub (number F20231020101828). All participants received a non-opposition form and thus agreed that their data would be used for research purposes.

### 2.2. Inclusion and Exclusion Criteria

To be included, patients were required to be over 18 years old; to have undergone botulinum toxin injection in their care pathway; to present with focal spastic hypertonia in at least 1 muscle of the upper or lower limbs, justifying the use of botulinum toxin; and to be able to provide answers with no cognitive disease for the evaluation of pain intensity and level of anxiety. All contraindications to toxin botulinum injection (e.g., myasthenia, pregnancy, breast-feeding, hypersensitivity to one of the substances in the product, infection at the injection site) and any pathological conditions not allowing for optimal use of the virtual reality helmet (e.g., blindness, major visual acuity disorders, deafness) were not included. Patients who were unable to retain the virtual reality headset during the procedure (e.g., appearance of adverse effects, patient wishing to stop during the procedure) were excluded from the study.

### 2.3. Procedure

**Figure 1:**
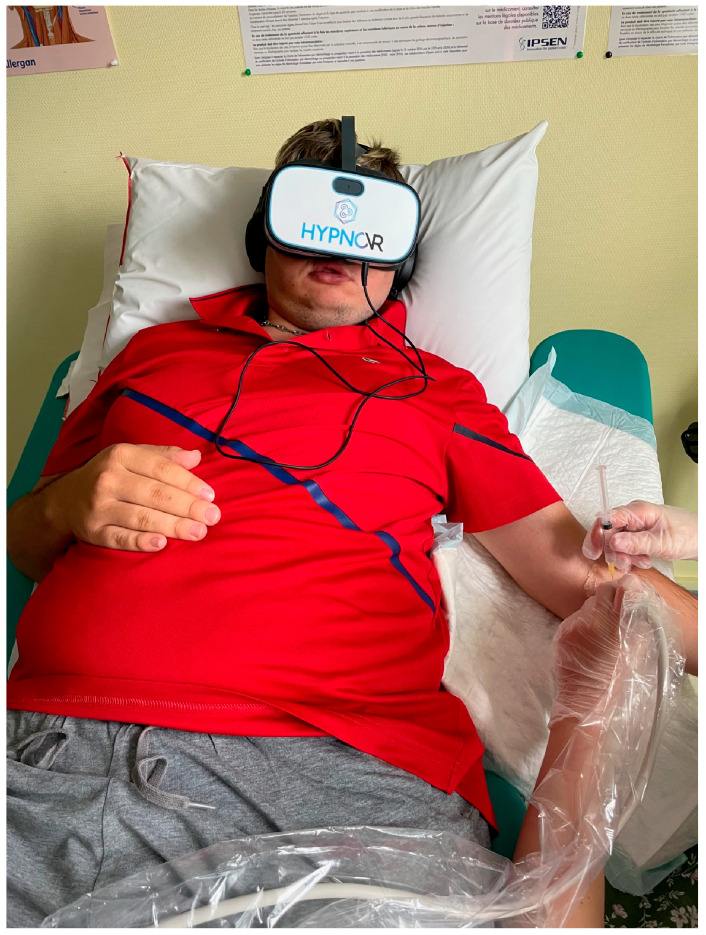
Patient during botulinum toxin injection with virtual reality headset.

**Figure 2:**
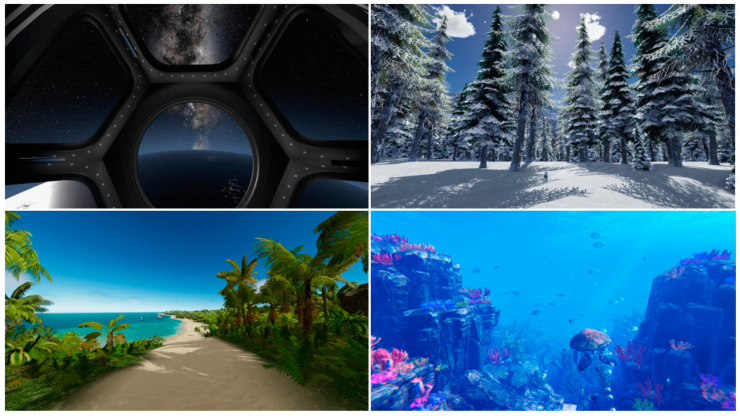
Virtual images on the headset (pictures from https://hypnovr.io/fr/, (accessed on 19 December 2023), all rights reserved).

The patients were informed about the procedure of VR utilization and consented to wear the device. The patients were comfortably seated on the examination table. The VR devices (HYPNOVR, Strasbourg, France, https://hypnovr.io/fr/produits/hypnovr/ (accessed on 19 December 2023)), combined with a headset (TaoTronic, model TT-BH085, reference 6972103466158, 21520 Yorba Linda Blvd, Suite G, Yorba Linda, CA 92887, USA), were set on the patients as comfortably as possible (Figure 1), and movies showing calm visual environments (walking on the beach, diving among colored fishes, or traveling in space) combined with relaxing music were displayed (Figure 2). The VR program consisted of a 2-min induction phase (before injection), 10–20 min of the main VR pathway (during injection), and a 2 min exit phase. Data were collected before and after the procedures. During the procedure, no signal was provided to the patient, optimizing the immersive quality of the virtual environment.

### 2.4. Outcomes

Pain intensity, considered as the primary endpoint, was determined using a numerical rating scale (NRS) ranging from 0 (no pain) to 10 (maximum imaginable pain) [32,33]. The patient reported the pain experienced during previous injections without VR before injection and the pain experienced in the current procedure with VR after the end of the procedure. In addition, perceived improvement was determined by the patient as a percentage of the added value of VR for pain intensity compared to the procedure without VR.

The level of anxiety, determined by the previous injection without VR and the current injection with VR, was determined using a numerical anxiety rating scale from 0 (no anxiety) to 10 (maximum imaginable anxiety) [34]. In addition, perceived improvement was determined by the patient as a percentage of the added value of VR on the level of anxiety compared to the procedure without VR.

The level of satisfaction was determined using an 11-point scale ranging from 0 (not satisfied at all) to 10 (very satisfied), which asked whether they agreed to reuse VR for the subsequent toxin botulinum injection and whether they would recommend VR to other patients.

The muscles targeted for injection, the method of localizing injection sites (ultrasound or electrostimulation), analgesic medication intake, use of additive analgesic for toxin injection, time of the day, and occurrence of adverse events were determined.

### 2.5. Statistical Analysis

The study population was characterized by age, sex, disease, and baseline pain intensity. Quantitative variables were described through either the mean and standard deviation or the median and interquartile range, depending on data normality. Categorical variables were described via numbers and percentages. Normality was verified using the Shapiro–Wilk test.

The pain intensity NRS and level of anxiety during the procedures with and without VR were compared using a Wilcoxon signed-rank test (paired test), since the variable was not normally distributed.

The mean and standard deviation of perceived improvement using VR and satisfaction with VR were also reported. R software version 4.2.0 was used for the analyses. All statistical tests were two-sided, and the significance threshold was fixed at 0.05.

## 3. Results

### 3.1. Participants

Twenty-one patients were identified over the 7-month inclusion period. Four patients were excluded, three due to incomplete data collection and one due to the occurrence of cybersickness with VR [35]. Overall, 17 patients were included and analyzed.

The patients’ characteristics are presented in Table 1. The mean participant age was 49.9 ± 10.6 years, with nine females (53%). Spasticity was subsequent to stroke for nine (52.9%) patients, cerebral palsy for three (17.6%), multiple sclerosis for two (11.8%), cervico-arthrosic myelopathy for one (5.9%), hereditary spastic paraplegia for one (5.9%), and meningitis for one (5.9%). One patient received MEOPA and one patient received EMLA^®^. One patient was treated with long-term TRAMADOL.

On average, 5.4 muscles were targeted per person, and the median number of injected muscles was 5. The different injection sites are presented in Table 2 and Table 3. The total number of injections per patient ranged from 1 to 10 injections.

### 3.2. Primary and Secondary Outcomes

**Figure 3:**
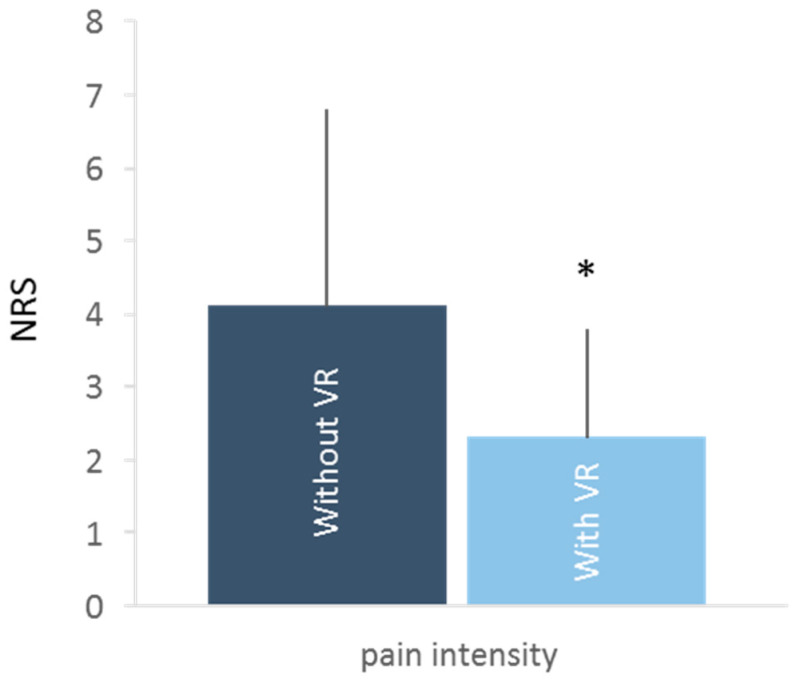
Mean NRS of pain intensity and its standard deviation during procedures with (light blue) and without VR (dark blue). * *p* < 0.05 between procedures with and without VR.

The pain intensity was significantly lower during the injection procedures with VR (2.3 ± 1.5) than those without VR (4.3 ± 2.7, *p* = 0.014) (Figure 3). The proportion of patients perceiving pain relief using VR was 76% (13/17).

The level of anxiety (NRS) was not significantly different between the injection with (1.3 ± 2.1) and that without VR (2.1 ± 3.0, *p* = 0.054) (Table 4). The proportion of patients perceiving anxiety reduction using VR was 29% (5/17).

Patients reported a mean subjective impression of improvement of 39.7 ± 30.9% for pain and 21.5 ± 25.0% for anxiety during the procedure with VR. The patients’ mean overall satisfaction was 7.9 ± 1.6 out of 10 (Table 2).

Regarding adverse events, only one patient (5.9%) experienced cyberkinetosis.

Fifteen patients (88.2%) agreed to reuse VR for a subsequent toxin botulinum injection, and all patients (100%) would recommend VR to other patients (Table 2).

## 4. Discussion

The objective of this study was to assess VR’s efficacy in reducing procedural pain and anxiety during botulinum toxin injection in adults presenting with spasticity. We showed that VR was able to significantly decrease procedural pain. Patients were very satisfied with the use of VR during the injections and agreed to reuse the VR helmet and recommend this approach to other patients.

In a systematic review including 18 studies, Smith et al. [25] reported that 12 studies demonstrated that VR led to significant pain reduction during painful procedures for burns, wounds, or injection. Similarly, in a systematic review including a meta-analysis, Mallari et al. [36] showed that VR could reduce acute procedural pain in adults. More specifically for botulinum injection procedures, using the Face, Legs, Activity, Cry, Consolability (FLACC) test for pain assessment, Chau et al. [28] reported a median score of 2.5 in 14 children treated for spasticity. In an adult population, we reported a pain intensity score of 2.3 with VR. Although previous research did not focus on procedural pain for botulinum toxin injection in adults, our study suggests that a non-invasive VR device can easily improve procedural injection, lasting for 3 months, and may ultimately enhance therapeutic adherence.

The main principle of VR is to provide strong and sufficient distraction to redirect attention initially focused on pain to a calm environment [37,38,39,40]. Thereby, VR can effectively modify sensory perceptions such as pain by monopolizing a high amount of attentional resources [41]. By having the patients’ attention compete between the VR environment and the pain, Rutter et al. [42] reported that VR led to the maintenance of an increased pain threshold and pain tolerance over 8 weeks (8 testing sessions) in 28 healthy participants during a cold-pressor task. In the current study, VR was applied with an immersive device, providing a high degree of immersion in a specific peaceful environment during one session of botulinum toxin injection [43,44,45]. While VR successfully managed chronic pain [46], the long-term and sustainable effects of VR in botulinum toxin injection in adults remain to be determined. In addition, combining hypnosis with VR could potentiate the effect of VR in managing procedural pain [47,48,49,50,51], and should be investigated in the future.

Although a positive effect of VR on anxiety has been reported in the literature [37,52], our results showed only a decreasing trend. That said, while we failed to observe a strong effect on anxiety, it is safe to assume that the level of anxiety at baseline (2.1) was too low for it to be significantly reduced in a population having already undergone several botulinum toxin injection procedures. An investigation of a population of patients receiving their very first injection would probably yield significantly decreased procedural anxiety related to botulinum toxin injection in adult populations.

Patients in the current study were very satisfied with VR (7.9 out of 10) and would agree to reuse VR for their next injection (88%), as well as to recommend VR for other patients (100%). Smith et al. [25] highlighted rare adverse events (8–10%) which were consistent with the single case of cybersickness observed in our study. The side effects observed during VR are considered as transient and reversible, making VR a safe approach to managing procedural pain in neurologic populations [35]. In addition, VR might be considered as a valuable alternative to medical therapies insofar as, in comparison with pharmacological management, it does not necessitate additional practitioners or costs other than the VR device itself. A medico-economic study should be conducted to validate this hypothesis.

This pilot study is associated with limitations. While the retrospective design in clinical routine provides real-world data, it entails potential bias connected with declarative assessment. As documented, pain perception could be modulated by temporal filtering, which could lead to a perception bias in our study. In addition, the results were not compared with those of a parallel control group. Future research relying on subjective pain intensity should investigate the potential additional benefit of VR during botulinum toxin injections using a crossover randomized controlled design. This approach ensures that patients report pain perception in both VR and non-VR conditions. The long-term added value of VR in botulinum toxin injection remains to be determined in a randomized controlled trial.

## 5. Conclusions

Our study showed that VR was useful for the management of pain related to botulinum toxin injection in adults, as patients were very satisfied with the device. In addition, they agreed to reuse VR for their next injection and to recommend this approach to other patients presenting with spasticity. While VR should be considered as an alternative treatment option to pharmacological approaches in botulinum toxin injection, a prospective randomized controlled trial with long-term follow-up and cost-effectiveness analysis is still required.

## Figures and Tables

**Table 1:** Patient characteristics.

Variables	Mean ± SD/*n* (%)
Age (years)	49.9 ± 10.6
Sex Male Female	
	8 (47.1%)
	9 (52.9%)
Disease diagnosis Stroke Cerebral palsy Multiple sclerosis Cervical myelopathy Hereditary spastic paraplegia Cerebrospinal meningitis	
	9 (52.9%)
	3 (17.6%)
	2 (11.8%)
	1 (5.9%)
	1 (5.9%)
	1 (5.9%)
Use of analgesic drugs No Yes	
	14 (82.4%)
	3 (17.6%)
Baseline pain intensity NRS (0–10) median (min–max)	0 (0–4)

NRS: numerical rating scale.

**Table 2:** Upper limb injection.

	Trapezius	Levator Scapulae	Pectoralis Major	Pectoralis Minor	Teres Major	Deltoid	Biceps Brachii	Brachialis	Brachioradialis	Triceps Brachii	Pronator Teres	Flexor Carpi Ulnaris	Flexor Carpi Radialis	Flexor Digitorum Superficialis	Flexor Digitorum Profundus	Flexor Pollicis Longus	Flexor Pollicis Brevis	Adductor Pollicis	Abductor Digiti Minimi of Hand	Interossei Dorsales
P1																				
P2			1											1	1	1				
P3																				
P4							1	1						1						
P5																				
P6	1	1						1						1	1			1		
P7																				
P8																				
P9			1		1			1						1	1					1
P10							1	1	1								1	1		
P11							1	1												
P12																				
P13												1	1	1	1					
P14								1				1	1						1	
P15								1						1	1					1
P16			1	1		1		1	1			1	1						1	
P17							1	1	1	1	1			1	1	1		1		1
%	1.1%	1.1%	3.4%	1.1%	1.1%	1.1%	4.5%	10.1%	3.4%	1.1%	1.1%	3.4%	3.4%	7.9%	6.7%	2.2%	1.1%	3.4%	2.2%	3.4%

P: Patient.

**Table 3:** Lower limb injection.

	Rectus Femoris	Adductor Longus	Tibialis Posterior	Gastrocnemius Medial Head	Gastrocnemius Lateral Head	Soleus	Flexor Digitorum Longus	Flexor Hallucis Longus
P1	1			1	1	1		
P2				1	1	1		
P3		1						
P4								
P5			1	1	1	1		
P6								
P7				1	1	1		
P8	1		1	1	1	1	1	
P9								
P10								
P11	1		1					
P12		1	1					
P13								
P14				1	1	1		
P15			1	1	1	1		1
P16								
P17								
%	3.4%	2.2%	5.6%	7.9%	7.9%	7.9%	1.1%	1.1%

P: Patient.

**Table 4:** Secondary outcome comparisons.

Variables	Without VR	With VR	*p*-Value
Anxiety during procedure	2.1 ± 3.0	1.3 ± 2.1	0.054
Perceived percentage of improvement Pain intensity Level of anxiety	-		
		39.7% ± 30.9%	
		21.5% ± 25.0%	
Patient satisfaction (0–10)	-	7.9 ± 1.6	
Agreed to reuse VR for next injection Yes No	-		
		15 (88.2%)	
		2 (11.8%)	
Does the patient recommend VR for other patients? Yes No	-		
		17 (100%)	
		0 (0%)	

## Data Availability

The data presented in this study are available upon request from the corresponding author. The data are not publicly available due to ethical considerations.
